# *GSTP1* and *ABCB1* Polymorphisms Predicting Toxicities and Clinical Management on Carboplatin and Paclitaxel‐Based Chemotherapy in Ovarian Cancer

**DOI:** 10.1111/cts.12937

**Published:** 2020-12-16

**Authors:** Amanda Canato Ferracini, Leisa Lopes-Aguiar, Gustavo Jacob Lourenço, Adriana Yoshida, Carmen Silva Passos Lima, Luis Otávio Sarian, Sophie Derchain, Deanna L. Kroetz, Priscila Gava Mazzola

**Affiliations:** ^1^ Postgraduate Program in Medical Sciences Faculty of Medical Sciences University of Campinas Campinas Brazil; ^2^ Department of Bioengineering and Therapeutic Sciences University of California San Francisco San Francisco California USA; ^3^ Laboratory of Cancer Genetics Faculty of Medical Sciences State University of Campinas Campinas Brazil; ^4^ Department of Obstetrics and Gynecology Faculty of Medical Sciences State University of Campinas Campinas Brazil; ^5^ Faculty of Pharmaceutical Sciences State University of Campinas Campinas Brazil

## Abstract

Variation in drug disposition genes might contribute to susceptibility to toxicities and interindividual differences in clinical management on chemotherapy for epithelial ovarian cancer (EOC). This study was designed to explore the association of *GST* and *ABCB1* genetic variation with hematologic and neurologic toxicity, changes in chemotherapy, and disease prognosis in Brazilian women with EOC. A total of 112 women with a confirmed histological diagnosis of EOC treated with carboplatin/paclitaxel were enrolled (2014–2019). The samples were analyzed by multiplex polymerase chain reaction (PCR) for the deletion of *GSTM1* and *GSTT1* genes. *GSTP1* (c.313A>G/rs1695) and *ABCB1* (c.1236C>T/rs1128503; c.3435C>T/rs1045642; c.2677G>T>A/rs2032582) single nucleotide polymorphisms (SNPs) were detected by real‐time PCR. Subjects with the *GSTP1* c.313A>G had reduced risk of anemia (odds ratio (OR): 0.17, 95% confidence interval (CI): 0.04–0.69, *P* = 0.01, dominant model) and for thrombocytopenia (OR: 0.27, 95% CI: 0.12–0.64, *P* < 0.01; OR 0.18, 95% CI 0.03–0.85, *P* = 0.03, either dominant or recessive model), respectively. The *GSTP1* c.313A>G AG genotype was associated with a lower risk of dose delay (OR: 0.35, 95% CI: 0.13–0.90, *P* = 0.03). The *ABCB1* c.1236C>T was associated with increased risk of thrombocytopenia (OR: 0.15, 95% CI: 0.03–0.82, *P* = 0.03), whereas *ABCB1* c.3435C>T had increased risk of grade 2 and 3 neurotoxicity (OR: 3.61, 95% CI: 1.08–121.01, *P* = 0.03) in recessive model (CC + CT vs. TT). This study suggests that *GSTP1* c.313A>G, *ABCB1* c.1236C>T, and c.3435C>T SNP detection is a potential predictor of hematological toxicity and neurotoxicity and could help predict the clinical management of women with EOC.

**Study Highlights**

**WHAT IS THE CURRENT KNOWLEDGE ON THE TOPIC?**

Variation in drug disposition genes encoding drug‐metabolizing enzymes and transporters might contribute to susceptibility to toxicities and interindividual differences in clinical management such as the need to delay, reduce, or discontinue treatment.

**WHAT QUESTION DID THIS STUDY ADDRESS?**

We studied the association of *GST* and *ABCB1* genetic variation with hematologic and neurologic toxicity, clinical management, and disease prognosis in Brazilian women with epithelial ovarian carcinoma (EOC) who undergo carboplatin and paclitaxel‐based chemotherapy.

**WHAT DOES THIS STUDY ADD TO OUR KNOWLEDGE?**

*GSTP1* c.313A>G is a potential predictor of anemia and thrombocytopenia and associated with a lower risk of dose delay during chemotherapy. In addition, *ABCB1* c.1236C>T and c.3435C>T is associated with a higher risk of thrombocytopenia and neurotoxicity.

**HOW MIGHT THIS CHANGE CLINICAL PHARMACOLOGY OR TRANSLATIONAL SCIENCE?**

The polymorphism detection could be a strategy to careful monitoring of patients at increased risk of toxicity and appropriate supportive therapy could decrease the need for changes in treatment, thus improving the likelihood of a beneficial treatment response in women with EOC.

Epithelial ovarian cancer (EOC) is the most common cause of gynecological cancer death, largely due to the advanced stage of the disease at the time of diagnosis.^1^ Standard first‐line treatment is cytoreductive surgery and subsequent chemotherapy using a combination of carboplatin and paclitaxel or neoadjuvant chemotherapy and residual tumor resection.^2^ Despite a high response rate to chemotherapy, ~ 70% of the women have a relapse within the subsequent 3 years.^3^ Platinum and taxane‐based chemotherapy are often associated with severe hematological toxicities, such as anemia, neutropenia, leukopenia, and thrombocytopenia.^4^ In addition, neuropathy is a dose‐limiting side effect of paclitaxel.^5^, ^6^ Interindividual differences in carboplatin and paclitaxel toxicity may be associated with polymorphisms in genes encoding drug‐metabolizing enzymes and transporters, including *GSTs* and ATP‐binding cassette (ABC) efflux transporters like *ABCB1*.^4^, ^7^, ^8^, ^9^

The GSTs are a family of phase II enzymes involved in detoxification of xenobiotics by conjugation reactions between glutathione and endogenous and exogenous electrophilic compounds, such as chemotherapeutic drugs, including the platinum agents. The *GST* family consists of several gene subfamilies of which *GSTM1*, *GSTT1*, and *GSTP1* are the most relevant for drug metabolism.^10^, ^11^ Functional GSTM1 and GSTT1 enzymes are directly related with the presence of the intact genes, because the absence of activity is the result of a 15 kb and 54 kb deletions that span the entire *GSTM1* and *GSTT1* genes (*GSTM1*‐null and *GSTT1*‐null genotypes), respectively. Consequently, individuals homozygous for the *GSTM1* or *GSTT1*‐null allele have a complete absence of GSTM1 and GSTT1 activity, whereas individuals with two copies of the *GSTM1* or *GSTT1* genes have reference protein levels.^12^, ^13^ There is some evidence that these deletion genotypes may play a role in toxicity, response to treatment, and survival in some cancers,^14^, ^15^, ^16^ including cancer of the ovary.^8^ In contrast to the commonly studied *GSTM1* and *GSTT1* genotypes, the *GSTP1* c.313A>G (rs1695) is an exonic single nucleotide polymorphism (SNP) that causes an amino acid substitution and results in an isoleucine to valine (Ile > Val) change at codon 105 of the enzyme. The highest level of GSTP1 activity is seen in individuals with the AA genotype (Ile/Ile) and is associated with increased toxicity in different carcinomas, but there are discordant results regarding the effect of *GSTP1* c.313A>G on treatment outcomes.^9^, ^17^, ^18^, ^19^, ^20^

Polymorphisms in *ABCB1* or multidrug resistance 1 may affect the function of P‐glycoprotein, a critical transporter for efflux of paclitaxel from cells.^21^, ^22^ Three SNPs in the coding region of *ABCB1* (c.1236C>T, rs1128503; c.3435C>T, rs1045642; and c.2677G>T>A, rs2032582) have been extensively studied.^23^, ^24^ These common *ABCB1* SNPs have been associated with toxicity during carboplatin and paclitaxel‐based chemotherapy, including increased risk of anemia in carriers of the c.1236C>T SNP, a more pronounced neutrophil decrease in patients carrying the c.3435C>T and c.2677G>T>A SNPs and increased risk of peripheral neuropathy associated with the c.3435C>T SNP.^18^, ^25^, ^26^ Similar to studies of *GST* polymorphisms, the associations of *ABCB1* genetic variation with treatment outcomes is inconsistent across studies.^27^, ^28^

Patients developing severe toxicities often require dose reduction, dose delay, or treatment interruption that require clinical interventions and may affect the disease prognosis.^4^ However, no study has been found so far focus on regarding the utility of polymorphisms in the management of chemotherapy and toxicities for ovarian cancer. The current study was designed to examine the association of *GST* and *ABCB1* genetic variants with hematologic and neurologic toxicities, clinical management on chemotherapy, and disease prognosis in Brazilian women with EOC.

## METHODS

### Study design, setting, and subjects

For this cohort study, the germline DNA samples and the respective files of women who attended the gynecologic oncology clinics at the Women’s Hospital of the University of Campinas (CAISM‐UNICAMP) between January 2014 and July 2019 and who were followed up through July 2020 were selected. Biological samples were stored at the Biobank number 56 from CAISM. This study was approved by the local research ethics committee (CAAE: 57829316.1.0000.5404). All procedures were carried out according to the Helsinki Declaration. All women signed the informed consent before being included in the biobank.

All women included had confirmed histological diagnosis of EOC classified according to the World Health Organization (WHO) criteria.^29^ Then, the cases were classified such as: low grade serous and others type I (endometrioid, clear cell, and mucinous carcinomas) and high grade serous and others type II (carcinosarcoma and undifferentiated carcinoma).^30^ The staging was performed according to the classification by the International Federation of Gynecology and Obstetrics (FIGO) for cancer of the ovary, fallopian tube, and peritoneum (stage I: tumor confined to ovaries or fallopian tube to stage IV: distant metastasis excluding peritoneal metastases)^31^; serum CA125 levels (UI/mL) were obtained at diagnosis for all cases. Clinicopathological data were obtained from the medical records and logged in a data collection form. According to the standard treatment protocol the women underwent six cycles of carboplatin/paclitaxel‐based chemotherapy. Carboplatin was dosed at a starting area under the plasma concentration‐vs. time curve (AUC) of 5–6 mg/mL/min, using Calvert’s formula. Paclitaxel was administered at a starting dose of 175 mg/m^2^.

The medical records were accessed to collect data about toxicity evaluations performed, as well as any clinical management possibly related to chemotherapy due to the patient’s health conditions or severe adverse events. The hematological toxicity was scored based on before each chemotherapy cycle to determine the nadir of anemia, leucopenia, neutropenia, and thrombocytopenia. In each woman, hematological toxicity and neurotoxicity were evaluated each cycle and graded by Common Terminology Criteria for Adverse Event (CTCAE) version 5.0.^32^ In this study, women who underwent at least one cycle of chemotherapy were included in the toxicity. The highest grade of toxicity over all courses within a patient was reported.

Chemotherapy was dose reduced (varied from 15% to 25% reduction), dose delay (characterized as any temporary suspension—at least 1 day—of a previously scheduled chemotherapy cycle) or treatment interruption (stopping of the originally prescribed standard chemotherapy protocol or permanent discontinuation of carboplatin or paclitaxel). In advanced cases, chemotherapy was continued beyond six cycles if the attending physician deemed the extension beneficial.

The secondary objectives were amended to include response to chemotherapy and progression‐free survival (PFS) and overall survival (OS), respectively, in these women. The platinum response was classified as recommended by Patch *et al*. (2015) in four categories: refractory, primary resistant, sensitive, and acquired resistance.^33^ Patients in the primary respondent group were restricted to those with progression or at least 6 months of follow‐up without disease. The PFS and OS time was estimated in months, from the date of diagnosis to the last follow‐up visit, recurrence, or any cause of death.^34^ The PFS was measured from the time of diagnosis until relapse, progressive disease, or last follow‐up, and OS from the time of diagnosis until any cause of death or last follow‐up.

### Genotyping

Genomic DNA was isolated from leukocytes by proteinase K treatment followed by extraction using phenol–chloroform.^35^ *GSTM1* and *GSTT1* were amplified by multiplex polymerase chain reaction (PCR) in the same reaction. β‐globin gene fragment (primer sense 5′ATACAATGTATCATG CCTCTTTGCACC3′; primer antisense 5′GTATTTTCC CAAGGTTTGAACTAGCTC3′), was amplified at the same PCR and used as a control of the DNA sample. Genotypes were analyzed by electrophoresis on a 2.0% agarose gel, and only those PCR signals were considered in which the corresponding β‐globin gene internal control was evident.^36^

Genotyping of SNPs *GSTP1* c.313A>G (rs1695, C___3237198_20), *ABCB1* c.1236C>T (rs1128503, C_7586662 _10), *ABCB1* c.3435C>T (rs1045642; C_7586657_20), and *ABCB1* c.2677G>T/A/(rs2032582, C_11711720C_30, C_11711720D_40) were determined by the StepOne Real‐Time PCR System on TaqMan Genotyper (Applied Biosystems, Califórnia, Estados Unidos) using commercially available predesigned TaqMan probes (Life Technologies). All genotyping was performed in a blinded fashion, including water as a negative control. As a quality control, 10% of all DNA samples were measured in duplicate. The PCR primers, restriction enzymes, and primer sequences for SNP assays are shown in **Table** **S1**.

### Statistical analysis

Hardy–Weinberg equilibrium was tested using the χ2 goodness‐of‐fit test. Continuous data were analyzed using the Mann–Whitney *U* test. The dominant (AA vs. AG + GG), and recessive (AA + AG vs. GG) models were utilized to target the *GSTP1* c.313A>G. The influence of the studied *ABCB1* SNPs (c.1236C>T, c.3435C>T, and c.2677G>T/A) was evaluated considering the genetic contrasts of dominant (CC vs. CT + TT for c.1236C>T and c.3435C>T, GG vs. GT/GA+/TT/AA/TA for 2677G>T/A), and recessive models (CC + CT vs. TT for 1236C>T and 3435C>T, GG + GT/GA vs. TT/TA/AA for c.2677G>T/A).^37^ Differences between toxicity and genotype were analyzed using the χ2 or Fisher’s exact test (categorical data). Multiple comparisons were performed only when a significant difference (*P* < 0.05) for a given SNP genotype set was found. We provided a multivariate analyses model using dominant and recessive models. Relevant clinical variables, such age, histological subtype, and FIGO stage with *P* < 0.01 are considered as covariates in multivariate analysis.^38^, ^39^ Data with *P* < 0.05 were included in multiple logistic regression models to adjust *P* values to obtain odd ratio (OR) values and 95% confidence intervals (95% CIs).

The Cox hazards model was used to identify variables that predicted PFS and OS and to obtain hazard ratio values and 95% CI. Variables for which *P* ≤ 0.10 in the univariate Cox analysis were included in the multivariate Cox analysis. Differences were significant when *P* < 0.05. All statistical tests were done using the R Environment for Statistical Computing Software and two‐sided significance was achieved when *P* < 0.05.^40^

## RESULTS

### Clinicopathological features and genotypes frequencies

The germline DNA samples from 112 women with EOC were available in the biobank, and all of them had complete data and were enrolled in the study. The median age of the patients was 58 years (range 22–87 years). At initial diagnosis, a total of 74 (66.1%) women had high‐grade serous carcinoma, undifferentiated or carcinosarcoma, CA‐125 levels were markedly elevated (median 1433.8 U/mL), 78 (69.6%) women were classified as stage III–IV, and 69 (61.6%) remained with postsurgery residual disease. The women received at least 1 cycle of carboplatin and paclitaxel chemotherapy and 85 (75.9%) women underwent at least 6 cycles of carboplatin and paclitaxel chemotherapy. The carboplatin/paclitaxel regimen was preferred as adjuvant treatment (*n* = 61, 54.5%; **Table** **1**). The null *GSTM1* and *GSTT1* genotypes were found for 41.1% and 26.8% of the women, respectively. Genotype and allele frequencies for *GSTP1* c.313A>G, *ABCB1* c.1236C>T *ABCB1* c.3435C>T, and *ABCB1* c.2677G>T/A SNPs in women are shown in **Table** **2**.

**Table 1:** Baseline clinical‐pathological characteristics among 112 women with EOC

Clinical features	Mean (SD) or *n* (%)
Age, years	58.1 ± 12.6
BMI, kg/m^2^	26.7 ± 4.9
Ethnicity	
Non‐white	8 (7.1)
White	104 (92.9)
Menopause	
No	23 (20.5)
Yes	89 (79.5)
Histological subtypes^a^	
LGS and other	38 (33.9)
HGS and other	74 (66.1)
CA 125 (U/mL)	1433.8 ± 2720.8
FIGO stage	
I + II	34 (30.4)
III + IV	78 (69.6)
Postsurgery residual disease	
No	43 (38.4)
Yes	69 (61.6)
Type of treatment	
Neoadjuvant	51 (45.5)
Adjuvant	61 (54.5)
Chemotherapy treatment	
Carboplatin	1 (0.9)
Carboplatin/paclitaxel	111 (99.1)

BMI, body mass index; EOC, epithelial ovarian cancer; FIGO, The International Federation of Gynecology and Obstetrics; HGS, high grade serous; LGS, low grade serous.

^a^ Histological subtypes: HGS and other: 66 (89.2%) cases of HGS carcinomas, 6 (8.1%) undifferentiated, 2 (3.1%) carcinosarcomas; LGS and other: 9 (23.7%) cases of endometrioid low grade carcinomas, 9 (23.7%) cases of clear cell carcinomas, 8 (21.1%) cases of mucinous, 6 (15.8%) cases of LGS carcinomas, and 6 (15.8%) cases of mixed carcinomas.

**Table 2:** Genotypes and alleles frequencies of SNPs among 112 women with EOC

Polymorphisms	rs ID	Homozygous wild type (%)	Heterozygous variant *n* (%)	Homozygous variant *n* (%)	MAF	HWE (p)
*GSTP1* c.313A>G	rs1695	AA 49 (43.8)	AG 44 (39.3)	GG 19 (17.0)	0.37	0.10
*ABCB1* c.1236C>T	rs1128503	CC 38 (33.9)	CT 57 (50.9)	TT 17 (15.2)	0.41	0.56
*ABCB1* c.3435C>T	rs1045642	CC 37 (33.0)	CT 60 (33.0)	TT 15 (13.4)	0.41	0.23
*ABCB1* c.2677G>T/A	rs2032582	GG 40 (35.7)	GT 50 (44.6) GA 5 (4.5)	TT 12 (10.7) TA 4 (3.6) AA 1 (0.9)	0.33^a^/0.03^b^	0.09

HWE, deviation from the Hardy–Weinberg equilibrium (χ2‐test); MAF, minor allele frequency; SNP, single‐nucleotide polymorphism.

^a^ MAF from genotype TT.

^b^ MAF from genotype AA.

### *GST* and *ABCB1* polymorphisms and chemotherapy‐induced toxicities

Thrombocytopenia grade 1 to grade 4 and neutropenia grade 3 and grade 4 was the most frequently reported severe hematologic toxicities (*n* = 36, 32.1% and *n* = 22, 19.7%, respectively), whereas 35 (31.3%) of participant showed severe grade (grade 2 and grade 3) for neurotoxicity **(** **Table** **S2** **)**. The *GSTM1*, *GSTP1* c.313A>G, *ABCB1* c.1236C>T, *ABCB1* c.3435C>T, and *ABCB1* c.G2677T/A polymorphisms met the *P* < 0.10 threshold in univariate analyses for toxicities **(** **Table** **S3** **)**. Of these, *GSTP1* c.313A>G, *ABCB1* c.1236C>T, and *ABCB1* c.3435C>T SNPs had *P* values < 0.05 for the association with at least one toxicity (**Table** **3**).

**Table 3:** Univariate analysis of SNPs in *GSTP1* and *ABCB1* and hematological and nonhematological toxicity

Polymorphism	Anemia			Thrombocytopenia			Neurotoxicity		
	G0‐G2 *n* (%)	G3‐G4 *n* (%)	*P*	G0 *n* (%)	G1–G4 *n* (%)	*P* value	G0‐G1 *n* (%)	G2‐G3 *n* (%)	*P* value
*GSTP1* c.313A>G									
AA	39 (79.6)	10 (20.4)	**0.04** a	26 (53.1)	23 (46.9)	**< 0.01** b	–	–	–
AG	42 (95.5)	2 (4.5)	33 (75.0)	11 (25.0)			–	–	–
GG	18 (94.7)	1 (5.3)	17 (89.5)	2 (10.5)			–	–	–
Dominant							–	–	–
AA	39 (79.6)	10 (20.4)	**0.01**	26 (53.1)	23 (46.9)	**< 0.01**	–	–	–
AG + GG	60 (95.2)	3 (4.8)	50 (79.4)	12 (20.6)			–	–	–
Recessive							–	–	–
AA + AG	81 (87.1)	12 (12.9)	0.46	59 (63.4)	34 (36.6)	**0.03**	–	–	–
GG	18 (94.7)	1 (5.3)	17 (89.5)	2 (10.5)			–	–	–
*ABCB1* c.1236C>T									
CC	–	–	–	26 (68.4)	12 (31.6)	**0.03** c	–	–	–
CT	–	–	–	43 (75.4)	14 (24.6)		–	–	–
TT	–	–	–	7 (41.2)	10 (58.8)		–	–	–
Dominant	–	–	–	–	–	–			
CC	–	–	–	26 (60.4)	12 (31.6)	1	–	–	–
TT + CT	–	–	–	50 (67.6)	24 (32.4)		–	–	–
Recessive	–	–	–	–	–	–			
CC + CT	–	–	–	69 (72.6)	26 (27.4)	**0.01**	–	–	–
TT	–	–	–	7 (41.2)	10 (58.8)		–	–	–
*ABCB1* c.3435C>T									
CC	–	–	–	–	–	–	29 (78.4)	8 (21.6)	**0.02** d
CT	–	–	–	–	–	–	42 (37.5)	18 (30.0)	
TT	–	–	–	–	–	–	6 (40.0)	9 (60.0)	
Dominant	–	–	–	–	–	–			
CC	–	–	–	–	–	–	29 (78.4)	8 (21.6)	0.14
TT + CT	–	–	–	–	–	–	48 (64.0)	27 (36.0)	
Recessive	–	–	–	–	–	–			
CC + CT	–	–	–	–	–	–	71 (73.2)	26 (26.8)	**0.01**
TT	–	–	–	–	–	–	6 (40.0)	9 (60.0)	

SNP, single‐nucleotide polymorphism.

G0–G4: grade; statistically significant differences are in bold; *P* values were calculated using the χ2/Fisher exact test.

^a^ AA vs. AG = 0.02.

^b^ AA vs. AG = 0.02 AA vs. GG = 0.005.

^c^ CC vs. TT = 0.05 CT vs. TT = 0.008.

^d^ CC vs. TT = 0.007 CT vs. TT = 0.03.

Severe anemia was found in 13 (11.6%) women and *GSTP1* c.313A>G AG genotype were less likely to have severe anemia (*P* = 0.04); only 23% of women with severe anemia in *GSTP1* c.313A>G (dominant model: AA vs. AG + GG) compared with 60% of subjects with no toxicity. Severe thrombocytopenia was found in 35 (31.2%) women and was also less frequent in women with the GSTP1 c.313A>G AG or GG genotype (*P* = 0.02 and *P* < 0.01, respectively); only 34% of subjects with grade 1 to grade 4 thrombocytopenia carried *GSTP1* c.313A>G (dominant model: AA vs. AG + GG) compared with 66% of carriers in the no toxicity group (*P* < 0.01). The *ABCB1* c.1236C>T (recessive model: CC + CT vs TT) was more common in women with thrombocytopenia (28%) compared with those with no toxicity (9%, *P* = 0.03). The *ABCB1* c.3435C>T was more common in women with neurotoxicity (25%) compared with those with good tolerance to chemotherapy (7%, *P* = 0.01; recessive model: CC + CT vs. TT). Univariate analysis results of *GST* null alleles and all genotyped SNPs and are shown in **Table** **S3**.

The association of anemia and thrombocytopenia with *GSTP1* c.313A>G, *ABCB1* c.1236C>T, and *ABCB1* c.3435C>T SNPs and key clinical features were evaluated by multivariate regression (as explained in the statistical methods) and are shown in **Table** **4**. The *GSTP1* c.313A>G AG genotype was associated with reduced risk of anemia (OR: 0.15, 95% CI: 0.03–0.82, *P* = 0.03). The significant result also was observed when *GSTP1* c.313A>G dominant model (AA vs. AG + GG) applied to the polymorphism (OR: 0.17, 95% CI: 0.04–0.69, *P* = 0.01). A lower risk of thrombocytopenia was associated with the *GSTP1* c.313A>G either dominant (AA vs. AG + GG; OR: 0.27, 95% CI: 0.12–0.64, *P* < 0.01) or recessive model (AA + AG vs. GG; OR: 0.18, 95% CI: 0.03–0.85, *P* = 0.03). The *ABCB1* c.1236C>T (recessive model: CC + CT vs. TT) was associated with increased risk of thrombocytopenia (OR: 0.15, 95% CI: 0.03–0.82, *P* = 0.03) whereas the carriers of the *ABCB1* c.3435C>T had increased risk of grade 2 and 3 neurotoxicity (OR: 3.61, 95% CI: 1.08–121.01, *P* = 0.03; recessive model: CC + CT vs. TT).

**Table 4:** Multivariate analysis of anemia and thrombocytopenia with *GSTP1* c.313A>G, *ABCB1* c.1236C>T, and *ABCB1* c.3435C>T polymorphisms

Polymorphism	Anemia		Thrombocytopenia		Neurotoxicity	
	OR (95% CI)	*P* adj	OR (95% CI)	*P* adj	OR (95% CI)	*P* adj
*GSTP1* c.313A>G						
AA	Reference		Reference		–	–
AG	**0.16 (0.03**–**0.84)**	**0.03**	**0.32 (0.12**–**0.82)**	**0.01**	–	–
GG	0.18 (0.02–1.64)	0.13	**0.11 (0.02**–**0.59)**	**< 0.01**	–	–
Dominant (AA vs AG + GG)	**0.17 (0.04**–**0.69)**	**0.01**	**0.27(0.12**–**0.64)**	**< 0.01**	–	–
Recessive (AA + AG vs GG)	0.34 (0.41–2.89)	0.32	**0.18 (0.03**–**0.85)**	**0.03**	–	–
*ABCB1* c.1236C>T						
CC	–	–	**Reference**		–	–
CT	–	–	0.77 (0.29–2.07)	0.61	–	–
TT	–	–	**3.63 (0.98**–**13.47)**	**0.05**	–	–
Dominant (CC vs. CT + TT)	–	–	1.04 (0.44–2.48)	0.93	–	–
Recessive (CC + CT vs. TT)	–	–	**3.50 (1.12**–**10.97)**	**0.03**	–	–
*ABCB1* c.3435C>T						
CC	–	–	–	–	Reference	
CT	–	–	–	–	1.41 (0.53–3.78)	0.49
TT	–	–	–	–	**4.54 (1.14**–**17.91)**	**0.03**
Dominant (CC vs. CT + TT)	–	–	–	–	1.79 (0.70–4.60)	0.22
Recessive (CC + CT vs. TT)	–	–	–	–	**3.61 (1.08**–**12.01)**	**0.03**

CI, confidence interval; OR, odds ratio.

Odds ratios was adjusted for age, histological subtypes, and International Federation of Gynecology and Obstetrics (FIGO); category bold values indicate statistically significant differences.

The association among *GSTP1* c.313A>G, *ABCB1* c.1236C>T, and *ABCB1* c.3435C>T SNPs and each of the three clinical managements of chemotherapy regimens were evaluated by multivariate regression, accounting for age, histological subtype, and FIGO stage. Among the 112 women who underwent chemotherapy, there were 18 (16.1%) who required dose reductions, 17 (15.2%) had dose delays, and 18 (16.1%) had treatment interruptions **(** **Table** **S2** **)**. These investigations into the clinical management of chemotherapy led us to the interesting observation that *GSTP1* c.313A>G AG genotype was associated with a lower risk of dose delay (OR: 0.35, 95% CI: 0.13–0.90, *P* = 0.03). The significant result also was observed when the dominant model (AA vs. AG + GG) applied to the polymorphism (OR: 0.36, 95% CI: 0.16–0.85, *P* = 0.02; **Table** **5**).

**Table 5:** Multivariate logistic regression analysis of clinical management of chemotherapy regimens with *GSTP1* c.313A>G, *ABCB1* c.1236C>T, and *ABCB1* c.3435C>T

	Dose reduction		Dose delay		Treatment interruption^a^	
	OR (95% CI)	*P* value	OR (95% CI)	*P* value	OR (95% CI)	*P* value
*GSTP1* c.313A>G						
AA	Reference		Reference		Reference	
AG	0.70 (0.28–1.57)	0.36	**0.35 (0.13**–**0.90)**	**0.03**	0.35 (0.10–1.23)	0.10
GG	0.31 (0.09–1.03)	0.06	0.43 (0.13–1.46)	0.18	0.60 (0.14–2.58)	0.49
Dominant (AA vs. AG + GG)	0.54 (0.25–1.16)	0.12	**0.36 (0.16**–**0.85)**	**0.02**	0.42 (0.15–1.20)	0.11
Recessive (AA + AG vs. GG)	0.38 (0.13–1.16)	0.08	0.68 (0.22–2.12)	0.51	0.93 (0.23–3.73)	0.92
*ABCB1* c.1236C>T						
CC	Reference		Reference		Reference	
CT	1.14 (0.36–3.64)	0.82	1.00 (0.28–3.54)	0.99	1.42 (0.28–7.26)	0.67
TT	3.33 (0.57––19.49)	0.18	1.54 (0.24–9.71)	0.64	2.03 (0.20–21.07)	0.55
Dominant (CC vs. CT + TT)	1.16 (0.39–3.45)	0.78	1.08 (0.32–3.63)	0.90	1.60 (0.32–7.90)	0.57
Recessive (CC + CT vs. TT)	3.08 (0.66–14.34)	0.15	1.71 (0.38–7.63)	0.48	1.73 (0.27–11.18)	0.56
*ABCB1* c.3435C>T						
CC	Reference		Reference		Reference	
CT	0.89 (0.28–2.77)	0.84	1.80 (0.51–6.31)	0.36	1.70 (0.34–8.44)	0.51
TT	0.51 (0.07–3.42)	0.49	0.96 (0.12–7.69)	0.98	1.12 (0.07–15.90)	0.93
Dominant (CC vs. CT + TT)	0.95 (0.32–2.83)	0.92	1.67 (0.48–5.79)	0.42	1.55 (0.31–7.75)	0.59
Recessive (CC + CT vs. TT)	0.52 (0.10 2.68)	0.43	0.52 (0.09–2.83)	0.45	0.63 (0.07–5.50)	0.68

CI, Confidence interval; OR, odds ratio.

Odds ratios was adjusted for age, histological subtypes, and International Federation of Gynecology and Obstetrics (FIGO); category bold values indicate statistically significant differences.

^a^ Cases of permanent discontinuation of the originally prescribed standard chemotherapy protocol (carboplatin and paclitaxel) or one of chemotherapeutic drug.

### *GST* and *ABCB1* polymorphisms and outcomes

The median follow‐up duration was 34 months (range: 0.6–73 months). At the last follow up, 42 (37.5%) women were alive without disease, 24 (21.4%) were alive with disease, and 45 (40.2%) had died from ovarian carcinoma. One (0.89%) woman died from neutropenic sepsis after her first chemotherapy cycle. A Cox regression model with age, histology, stage of disease, *GSTP1* c.313A>G, *ABCB1* c.1236C>T, and *ABCB1* c.3435C>T SNPs was developed to assess factors associated with PFS and OS.

There was no statistically significant difference between either polymorphism models and PFS or OS; only age and FIGO stage predicted survival (**Table** **S4** **)**. Toxicity or clinical management were not considered in the multivariate survival model because they were not significant in univariate analysis (data not shown). No statistically significant difference was found between response to chemotherapy (sensitive vs. resistant) and the studied polymorphisms (**Table** **S5** **)**.

## DISCUSSION

In this study, hematological toxicity and neurotoxicity were a major complication in carboplatin and paclitaxel‐based chemotherapy in women with EOC. Although several features are associated with this adverse event, including advanced stage, polymorphisms in drug disposition genes may also contribute to variation in these toxicities. The *GSTP1* c.313A>G (dominant model: AA + AG vs. GG) was associated with reduced risk of anemia and thrombocytopenia. In addition, the *ABCB1* c.1236C>T and *ABCB1* c.3435C>T (recessive model: CC + CT vs. TT) genotype had increased risk of thrombocytopenia and grade 2 and 3 neurotoxicity, respectively. In EOC chemotherapy, clinical management alterations may be required. In the current study, lower risk of dose delay was associated with the *GSTP1* c.313A>G (dominant model: AA vs. AG + GG).

The relationship of carboplatin and paclitaxel‐based chemotherapy with hematological toxicity is widely recognized. To assess the association between genotype and chemotherapy‐induced toxicities, the toxicities were classified as having good or poor tolerance to treatment; grades 3 and 4 of anemia, leukopenia, and neutropenia, any grade of thrombocytopenia and grades 2 and 3 of neurotoxicity were considered as grades of toxicity considered severe for women.^41^ The frequencies of hematological toxicities, such as anemia, leukopenia, neutropenia, and thrombocytopenia, were variable in previous studies.^4^, ^14^, ^17^, ^18^, ^19^, ^26^, ^27^, ^41^, ^42^ The poor tolerance of thrombocytopenia (grade 1‐above) differs from the other hematological toxicities because it was classified according to platelet counts < 75,000/mm^3^ and literature.^19^, ^42^, ^43^ Perhaps different frequencies of hematological toxicities are related to the timing of blood sample collection.^14^, ^41^ In our study, hematological toxicity was scored based on samples collected before each cycle of chemotherapy (about 21 days) to determine the recovery of red blood cells, neutrophils, and platelets.

Previous studies have demonstrated the association between chemotherapy‐related severe anemia and thrombocytopenia and the *GSTP1* c.313A>G polymorphism in patients who received carboplatin and paclitaxel‐based chemotherapy. Yoshihama *et al*. in 2018 have demonstrated that the “A” allele had a significantly higher risk of severe hematological toxicity (anemia grade 3, neutropenia grade 4, and thrombocytopenia grade 3) than the “G” allele in women with EOC receiving carboplatin and paclitaxel.^20^ Similar findings were reported in other studies; in 118 patients with ovarian cancer treated with platinum‐based chemotherapy, the *GSTP1* c.313 A>G, the A allele was a significant risk factor for grade 3 or 4 hematological toxicity. Otherwise, in 97 patients with advanced non‐small cell lung cancer with the variant alleles at *GSTP1* c.313A>G, have notably lower risk of anemia.^17^, ^44^ However, when data from 12 individual studies were compiled in a meta‐analysis of 1,657 men and women undergoing platinum chemotherapy, the *GSTP1* c.313A>G polymorphism was not significantly associated with thrombocytopenia.^19^

A significant problem with chemotherapy toxicity is the need to delay, reduce, or discontinue treatment. The *GSTP1* c.313A>G AG genotype was associated with a lower risk of dose delay. There is no established effect of this synonymous variant on GST function. Far reaching our knowledge, there are no studies focusing association of this SNP with dose delay. GSTP1 appears to have functional roles that extend beyond phase II drug metabolism. The conjugation of glutathione with platinum decreased the amount of free intracellular drug and the cytotoxic potential of platinum metabolites because increased water solubility favors their elimination from the body.^45^ Individuals with the *GSTP1* c.313A>G AA genotype are predicted to have decreased gene function that confers susceptibility to inflammatory symptoms.^46^, ^47^ The decrease of *GSTP1* activity in women with *GSTP1* c.313A>G AA genotype may result in a higher exposure to carboplatin and potentially increased drug toxicity because of reduced GST‐catalyzed reactions and reduced metabolism of platinum‐based drugs and consequently a clinical management may require. If confirmed in additional studies, the *GSTP1* c.313A>G might have utility in predicting the need for treatment modifications. Careful monitoring of patients at increased risk of toxicity and appropriate supportive therapy could decrease the need for dose delay in treatment, thus improving the likelihood of a beneficial treatment response.

In multivariate analysis *ABCB1* c.1236C>T and *ABCB1* c.3435C>T recessive model (CC + CT vs. TT) had increased risk of thrombocytopenia and grades 2 and 3 neurotoxicity, respectively. In a systematic review by Frederiks *et al*. (2015) there was no evidence for an association between *ABCB1* polymorphisms and thrombocytopenia in EOC.^48^ The *ABCB1* c.1236C>T homozygous variant genotypes have been associated with less neutropenia in women with ovarian carcinoma receiving either carboplatin plus paclitaxel combination therapy or paclitaxel monotherapy.^27^ In contrast, the *ABCB1* c.3435C>T and c.2677G>T/A polymorphisms (dominant model) were associated with more pronounced neutropenia in another study.^26^ Variant alleles at *ABCB1* c.1236C>T have been associated with higher risk of anemia.^18^ These discordant results support larger studies to define the role of *ABCB1* genetic variation in toxicity associated with carboplatin and paclitaxel chemotherapy. Otherwise, *ABCB1* variants have been reported to be associated with both increased and decreased risks of peripheral neuropathy.^25^, ^45^, ^46^, ^47^ In a study conducted by Sissung *et al*. (2006) involving 22 patients experiencing peripheral neuropathy, showed a trend toward an increased risk of neurotoxicity for individuals carrying at least one variant allele of *ABCB1* c.3435C>T.^45^ In a recent study conducted by Zhong *et al*. (2019), they suggested that *ABCB1* c.3435C>T TT and TC genotype in patients with lung cancer were more likely to have neuritis in taxane treatment.^25^ In contrast, there are studies showing that *ABCB1* c.3435C>T TT variant does not justify the significant interindividual variability in paclitaxel pharmacokinetics^46^ and did not associate with the occurrence of neurotoxicity in their patients with breast or ovarian cancer.^47^ These discordant results support larger studies to define the role of *ABCB1* genetic variation in neurotoxicity associated with carboplatin and paclitaxel. Although our result suggests that *ABCB1* genetic variants may be differentially expressed in the variants alleles, there is no established effect of this synonymous variant on P‐glycoprotein function or *ABCB1* expression, so the mechanism of this association is unclear.

Some limitations in the current study warrant discussion. Although the *ABCB1* SNPs haplotype is a better predictor of P‐glycoprotein‐related drug effects,^27^, ^49^ *ABCB1* SNPs haplotypes were no formed in our study population showing one of the limitations. Another limitation, as with most pharmacogenetic studies, was that SNPs with low minor allele frequency require a large sample size to achieve adequate power for statistical tests. In current study, we also did not have an external validation. Independently, we had access to DNA samples from patients with different histological subtypes and robust toxicity data, further external validations in independent cohorts to confirm the associations between SNPs, and toxicities in EOC women outside of a single institution are important before implementing prediction models in clinical practice.^50^ Otherwise, this study was carried out in the Brazilian reference center of our health district, which allowed all women to be diagnosed and treated by the same team, reducing the possibility that the findings were biased by population structure. Because survival of women with advanced ovarian cancer is poor, regardless of treatment, small changes in prognosis factors like genetic polymorphisms and clinical management can be difficult to detect.

In conclusion, the present study suggests that *GSTP1* c.313A>G is a potential predictor of anemia and thrombocytopenia and associated with a lower risk of dose delay during chemotherapy. In addition, *ABCB1* c.1236C>T and c.3435C>T is associated with a higher risk of thrombocytopenia and neurotoxicity. Polymorphism detection could help predict the clinical management of women with EOC who undergo carboplatin and paclitaxel‐based chemotherapy.

## Conflict of Interest

All other authors declared no competing interests for this work.

## Funding

This study was partially funded by a fellowship from Coordination for the Improvement of Higher Education Personnel (CAPES) number 01P‐3368/2017 and from São Paulo Research Foundation (FAPESP) grant 2016/22335‐2, 2018/00070‐2 (ACF). SD received a Grant by 303742/2018‐6 from Brazilian National Research Council (CNPq) and Brazil and Print Capes 88881.310833/2018‐01.

## Disclaimer

As Deputy Editor‐in‐Chief of *Clinical and Translational Science*, Deanna L. Kroetz was not involved in the review or decision process for this paper.

## Author Contributions

A.C.F., S.D., D.L.K., and P.G.M. wrote the manuscript. A.C.F., S.D., and P.G.M. designed the research. A.C.F. performed the research. A.C.F., L.L.A., G.J.L., A.Y., L.O.S., S.D., D.L.K., and P.G.M. analyzed the data. G.J.L., C.S.P.L., S.D., and P.G.M. contributed new reagents and analytical tools.

## Supporting information

Table S1

Table S2

Table S3

Table S4

Table S5
