# Supplementary material for: GSTP1 and ABCB1 Polymorphisms Predicting Toxicities and Clinical Management on Carboplatin and Paclitaxel‐Based Chemotherapy in Ovarian Cancer
Source: Clin Transl Sci. 2020 Dec 16;14(2):720–8. doi: 10.1111/cts.12937 (PMC7993324; doi:10.1111/cts.12937)
Supplement: Supplementary file 1 — Table S1 [file CTS-14-720-s002.pdf]

Table S1. PCR primers, restriction enzymes and primer sequences for SNP assays.

Polymorphisms	rs ID	Enzyme	Primer sequences
GSTM1	gene deletion	-	5'-CTGCCCTACTTGATTGATGGG-3'<br>5'-CTGGATTGTAGCAGATCATGC-3'
GSTT1	gene deletion	-	5'-TTCCTTACTGGTCCTCACATCTC-3'<br>5'-TCACCGGATCATGGCCAGCA-3'
GSTP1 c.313A>G	rs1695	*Alw*26I	5'-ACCCCAGGGCTCTATGGGAA-3'<br>5'-TGAGGGCACAAGAAGCCCCT-3'
ABCB1 c.1236C>T	rs1128503	*Eco0109I*	5-'TTCACTTCAGTTACCCATC -3'<br>5-'TCTTTGTCACTTTATCCAGC -3'
ABCB1 c.3435C>T	rs1045642	*MboI*	5'-GATCTGTGAACTCTTGTTTTC-3'<br>5-'CTTGTTTTCAGCTGCTTGATGGCAA-3'
ABCB1 c.2677G>T/A	rs2032582	*BanI*	5'-TGCAGGCTATAGGTTCCA GG-3'<br>5'-TTTAGTTTGACTCACCTTCCCG-3'

SNP, single-nucleotide polymorphism.
