# Supplementary material for: GSTP1 and ABCB1 Polymorphisms Predicting Toxicities and Clinical Management on Carboplatin and Paclitaxel‐Based Chemotherapy in Ovarian Cancer
Source: Clin Transl Sci. 2020 Dec 16;14(2):720–8. doi: 10.1111/cts.12937 (PMC7993324; doi:10.1111/cts.12937)
Supplement: Supplementary file 2 — Table S2 [file CTS-14-720-s005.pdf]

Table S2. Hematological toxicity, neurotoxicity and chemotherapy management alterations rates

**Features**	**n (%)**
**Toxicities**	
**Anemia**	
G0 - G2	99 (88.3)
G3 - G4	13 (11.7)
**Leukopenia**	
G0 - G2	101 (90.2)
G3 - G4	11 (9.8)
**Neutropenia**	
G0 - G2	90 (80.3)
G3 - G4	22 (19.7)
**Thrombocytopenia**	
G0	76 (67.9)
G1 - G4	36 (32.1)
**Neurotoxicity**	
G0 - G1	77 (68.7)
G2 - G3	35 (31.3)
**Chemotherapy management alterations**	
**Dose reduction**	
No	94 (83.9)
Yes	18 (16.1)
**Dose delay**	
No	95 (84.8)
Yes	17 (15.2)
**Treatment interruption**	
No	94 (83.9)
Yes	18 (16.1)
