# Supplementary material for: GSTP1 and ABCB1 Polymorphisms Predicting Toxicities and Clinical Management on Carboplatin and Paclitaxel‐Based Chemotherapy in Ovarian Cancer
Source: Clin Transl Sci. 2020 Dec 16;14(2):720–8. doi: 10.1111/cts.12937 (PMC7993324; doi:10.1111/cts.12937)
Supplement: Supplementary file 3 — Table S3 [file CTS-14-720-s003.pdf]

Table S3. Association of hematologic and non-hematologic toxicities with *GST* and *ABCB1* gene polymorphisms in 112 women with ovarian carcinoma

Polymorphisms	Hematological												Non-hematological		
	Anemia			Leukopenia			Neutropenia			Thrombocytopenia			Neurotoxicity		
	G0-G2 n (%)	G3-G4 n (%)	*p*	G0-G2 n (%)	G3-G4 n (%)	*p*	G0-G2 n (%)	G3-G4 n (%)	*p*	G0 n (%)	G1-G4 n (%)	*p*	G0-G1 n (%)	G2-G3 n (%)	*p*
GSTM1*															
Null	41 (89.1)	5 (10.9)	0.79	44 (95.7)	2 (4.3)	**0.09**	39 (84.8)	7 (15.2)	0.29	21 (67.4)	15 (32.6)	0.98	29 (63.0)	17 (37.0)	0.24
Present	56 (87.5)	8 (12.5)		55 (85.9)	9 (14.1)		49 (76.6)	15 (23.4)		43 (67.2)	21 (32.8)		47 (73.4)	17 (26.6)	
GSTT1*															
Null	25 (83.3)	5 (16.7)	0.33	27 (90.0)	3 (10.0)	1.0	23 (76.7)	7 (23.3)	0.59	20 (66.7)	10 (33.3)	0.93	21 (70.0)	9 (30.0)	0.90
Present	72 (90.0)	8 (10.0)		72 (90.0)	8 (10.0)		65 (81.3)	15 (18.8)		54 (67.5)	26 (32.5)		55 (68.8)	25 (31.3)	
***GSTP1* c.313A>G**															
AA	39 (79.6)	10 (20.4)		41 (83.7)	8 (16.3)		39 (79.6)	10 (20.4)		26 (53.1)	23 (46.9)		37 (75.5)	12 (24.5)	
AG	42 (95.5)	2 (4.5)	**0.04**	42 (95.5)	2 (4.5)	0.12	37 (84.1)	7 (15.9)	0.62	33 (75.0)	11 (25.0)	**<0.01**	27 (61.4)	17 (38.6)	0.34
GG	18 (94.7)	1 (5.3)		18 (94.7)	1 (5.3)		14 (73.7)	5 (26.3)		17 (89.5)	2 (10.5)		13 (68.4)	6 (31.6)	
Dominant															
AA	60 (95.2)	3 (4.8)	**0.01**	60 (95.2)	3 (4.8)	0.06	51 (81.0)	12(19.0)	1	50 (79.4)	10(20.6)	**<0.01**	40 (63.5)	23 (36.5)	0.22
AG+GG	39 (79.6)	10 (20.4)		41 (83.7)	8 (16.3)		39 (79.6)	10 (20.4)		26 (53.1)	23 (46.9)		37 (75.5)	12 (24.5)	
Recessive															
AA+AG	81 (87.1)	12 (12.9)	0.46	83 (89.2)	10 (10.8)	0.69	76 (81.7)	17 (18.3)	0.52	59 (63.4)	34 (36.6)	**0.03**	64 (68.8)	29 (31.2)	1
GG	18 (94.7)	1 (5.3)		18 (94.7)	1 (5.3)		14 (73.7)	5 (26.3)		17 (89.5)	2 (10.5)		13 (68.4)	6 (31.6)	
***ABCB1* c.1236 C>T**															
CC	36 (94.7)	2 (5.3)		36 (94.7)	2 (5.3)		30 (89.9)	8 (21.1)		26 (68.4)	12 (31.6)		28 (73.7)	10 (26.3)	
CT	50 (87.7)	7 (12.3)	0.14	49 (86.0)	8 (14.0)	0.31	45 (78.9)	12 (21.1)	0.67	43 (75.4)	14 (24.6)	**0.03**	41 (71.9)	16 (28.1)	0.11
TT	13 (76.5)	4 (23.5)		16 (94.1)	1 (5.9)		15(88.2)	2 (11.8)		7 (41.2)	10 (58.8)		8 (47.1)	9 (52.9)	
Dominant															
CC	63 (85.1)	11 (14.9)	0.21	65 (87.8)	9 (12.2)	0.32	60 (81.1)	14 (18.9)	0.80	50 (67.6)	24 (32.4)	1	49 (66.2)	25 (33.8)	0.52
CT+TT	36 (94.7)	2 (5.3)		36 (94.7)	2 (5.3)		30 (78.9)	8 (21.1)		26 (60.4)	12 (31.6)		28 (73.7)	10 (26.3)	
Recessive															
CC+CT	86 (90.5)	9 (9.5)	0.11	85 (89.5)	10 (10.5)	0.69	75 (78.9)	20 (21.1)	0.51	69 (72.6)	26 (27.4)	**0.01**	69 (72.6)	26 (27.4)	0.05
TT	13 (76.5)	4 (23.5)		16 (94.1)	1 (5.9)		15 (88.2)	2 (11.8)		7 (41.2)	10 (58.8)		8 (47.1)	9 (52.9)	

***ABCB1* c.3435 C>T**															
CC	35 (94.6)	2 (5.4)		36 (97.3)	1 (2.7)		30 (81.1)	7 (18.9)		27 (73.0)	10 (27.0)		29 (78.4)	8 (21.6)	
CT	52 (86.7)	8 (13.3)	0.27	52 (86.7)	8 (13.3)	0.21	47 (78.3)	13 (21.7)	0.76	42 (70.0)	18 (30.0)	0.16	42 (37.5)	18 (30.0)	**0.02**
TT	12 (80.0)	3 (20.0)		13 (86.7)	2 (13.3)		13 (86.7)	2 (13.3)		7(46.7)	8 (53.3)		6 (40.0)	9 (60.0)	
Dominant															
CC	64 (85.3)	11 (14.7)	0.21	65 (86.7)	10 (13.3)	0.09	60 (80.0)	15 (20.0)	1	49 (65.3)	26 (34.7)	0.52	48 (64.0)	27 (36.0)	0.14
TT+CT	35 (94.6)	2 (5.4)		36 (97.3)	1 (2.7)		30 (81.1)	7 (18.9)		27 (73.0)	10 (27.0)		29 (78.4)	8 (21.6)	
Recessive															
CC+CT	87 (89.7)	10 (10.3)	0.38	88 (90.7)	9 (9.3)	0.64	77 (79.4)	20 (20.6)	0.73	69 (71.1)	28 (32.1)	0.07	71 (73.2)	26 (26.8)	**0.01**
TT	12 (80.0)	3 (20.0)		13 (86.7)	2 (13.3)		13 (86.7)	2 (13.3)		7 (46.7)	8 (53.3)		6 (40.0)	9 (60.0)	
***ABCB1* c.2677 G>T/A**															
GG	39 (97.5)	1 (2.5)		38 (95.0)	2 (5.0)		33 (82.5)	7 (17.5)		28 (70.0)	12 (30.0)		30 (75.0)	10 (25.0)	
GT/GA	46 (83.6)	9 (16.4)	**0.08**	48 (87.3)	7 (12.7)	0.44	43 (78.2)	12 (21.8)	0.85	39 (70.9)	16 (29.1)	0.36	37 (67.3)	18 (32.7)	0.46
TT/TA/AA	14 (82.4)	3 (17.6)		15 (88.2)	2 (11.8)		14(82.4)	3 (17.6)		9 (52.9)	8 (47.1)		10 (58.8)	7 (41.2)	
Dominant															
GG	60 (83.3)	12 (16.7)	**0.03**	63 (87.5)	9 (12.5)	0.32	57 (79.2)	15 (20.8)	0.81	48 (66.7)	24 (33.3)	0.83	47 (65.3)	25 (34.7)	0.40
GT/GA+TT/TA/AA	39 (97.5)	1 (2.5)		38 (95.0)	2 (5.0)		33 (82.5)	7 (17.5)		28 (70.0)	12 (30.0)		30 (75.0)	10 (25.0)	
Recessive															
GG+GT/GA	85 (89.5)	10 (10.5)	0.41	86 (90.5)	9 (9.5)	1	76 (80.0)	19 (20.0)	1	67 (70.5)	28 (29.5)	0.17	67 (70.5)	28 (29.5)	0.39
TT/TA/AA	14 (82.4)	3 (17.6)		15 (88.2)	2 (11.8)		14 (82.4)	3 (17.6)		9 (52.9)	8 (47.1)		10 (58.8)	7 (41.2)	

(G0-G4): grade; *The number of women evaluated (n = 110) differs from the total (n = 112), due to an insufficient amount of DNA to perform the genotyping by the multiplex polymerase chain reaction (PCR) method; (n): number of patients; (G0-G4): grade; statistically significant differences are in bold, *p* values were calculated using the Chi square/Fisher exact test.
