# Supplementary material for: GSTP1 and ABCB1 Polymorphisms Predicting Toxicities and Clinical Management on Carboplatin and Paclitaxel‐Based Chemotherapy in Ovarian Cancer
Source: Clin Transl Sci. 2020 Dec 16;14(2):720–8. doi: 10.1111/cts.12937 (PMC7993324; doi:10.1111/cts.12937)
Supplement: Supplementary file 4 — Table S4 [file CTS-14-720-s001.pdf]

Table S4. The relationship between survival and clinical and genetic features model using a Cox regression in 112 EOC women

**Features**	**Progression free survival**			**Overall survival**		
	**Events/total**	**HR (95% CI)**	***p***	**Events/total**	**HR (95% CI)**	***p***
**Age (years ± SD)**	60.3± 11,9/ 58.1 ± 12.6	1.02 (1.00-1.04)	**0.04**	61.8 ± 12,9/ 58.1 ± 12.6	1.04 (1.01-1.07)	**<0.01**
**Histological subtypes§**						
LGS and other	16/38	Reference	0.41	10/38	Reference	0.97
HGS and other	58/74	0.75 (0.37-1.51)		36/74	0.98 (0.37-2.58)	
**FIGO Stage**						
I+II	**12/34**	**Reference**	**<0.01**	**8/34**	**Reference**	**0.04**
III + IV	**62/78**	**4.04 (1.95-8.38)**		**38/78**	**2.94 (1.07-8.11)**	
***GSTP1* c.313A>G**						
AA	35/49	Reference		22/49	Reference	
AG	26/44	0.91 (0.53-1.55)	0.72	26/44	0.62 (0.32-1.24)	0.18
GG	13/19	0.91 (0.45-1.84)	0.80	8/19	0.68 (0.29-1.59)	0.37
***ABCB1* c.1236C>T**						
CC	25/38	Reference		15/38	Reference	
CT	36/57	0.99 (0.50-1.99)	0.99	22/57	1.01 (0.37-2.70)	0.99
TT	13/17	0.98 (0.37-2.57)	0.97	9/17	1.01 (0.29-3.47)	0.98
***ABCB1* c.3435C>T**						
CC	26/37	Reference		14/37	Reference	
CT	38/60	0.93 (0.48-1.79)	0.82	26/60	1.39 (0.52-3.68)	0.51
TT	10/15	0.56 (0.19-1.60)	0.28	6/15	1.01 (0.30-3.47)	0.95

EOC: epithelial ovarian cancer SD: standard deviation; n: number of patients; §Histological subtypes: HGS (high grade serous) and other: 66 (89.2%) cases of high grade serous carcinomas, 6 (8.1%) undifferentiated, 2 (3.1%) carcinosarcomas,; LGS (low grade serous) and other: 9 (23.7%) case of endometrioid low grade carcinomas, 9 (23.7%) clear cell carcinomas, 8 (21.1%) mucinous, 6 (15.8%) low grade serous carcinomas and 6 (15.8%) mixed carcinoma; FIGO: The International Federation of Gynecology and Obstetrics. statistically significant differences are in bold; HR=hazard ratio; CI=Confidence interval.
