# Supplementary material for: GSTP1 and ABCB1 Polymorphisms Predicting Toxicities and Clinical Management on Carboplatin and Paclitaxel‐Based Chemotherapy in Ovarian Cancer
Source: Clin Transl Sci. 2020 Dec 16;14(2):720–8. doi: 10.1111/cts.12937 (PMC7993324; doi:10.1111/cts.12937)
Supplement: Supplementary file 5 — Table S5 [file CTS-14-720-s004.pdf]

Table S5. The relationship between genotypes and chemotherapy response

**Polymorphism**	**Sensitive**	**Resistant**	***p***
GSTM1*			
Null	24 (52.2)	22 (47.8)	0.17
Present	41 (65.1)	22 (34.9)	
GSTT1*			
Null	22 (73.3)	8 (26.7)	0.07
Present	43 (54.4)	36 (45.6)	
***GSTP1* c.313A>G**			
AA	29 (59.2)	20 (40.8)	0.42
AG	28 (65.1)	15 (34.9)	
GG	9 (47.4)	10 (52.6)	
Dominant			
AA	37 (59.7)	25 (40.3)	0.56
AG+GG	29 (59.2)	20 (40.8)	
Recessive			
AA+AG	57 (62.0)	35 (38.0)	0.18
GG	9 (47.4)	10 (52.6)	
***ABCB1* c.1236C>T**			
CC	23 (62.2)	14 (37.8)	0.53
CT	35 (61.4)	22 (38.6)	
TT	8 (47.1)	9 (52.9)	
Dominant			
CC	23 (62.2)	14 (37.8)	0.42
CT+TT	43 (58.1)	31 (41.9)	
Recessive			
CC+CT	58 (61.7)	36 (38.3)	0.19
TT	8 (47.1)	9 (52.9)	
***ABCB1* c.3435C>T**			
CC	25 (67.6)	12 (32.4)	0.43
CT	32 (54.2)	27 (45.8)	
TT	9 (60.0)	6 (40.0)	
Dominant			
CC	25 (67.6)	12 (32.4)	0.15
CT+TT	41 (55.4)	33 (44.6)	
Recessive			
CC+CT	57 (59.4)	39 (40.6)	0.59
TT	9 (60.0)	6 (40.0)	
***ABCB1* c.2677G>T/A**			
GG	25 (62.5)	15 (37.5)	0.80
GT/GA	32 (59.3)	22 (40.7)	
TT/TA/AA	9 (52.9)	8 (41.7)	
Dominant			
GG	25 (62.5)	15 (37.5)	0.39
GT/GA+TT/TA/AA	41 (57.7)	30 (42.3)	

Recessive			
GG+GT/GA	57 (60.6)	37 (39.4)	0.37
TT/TA/AA	9 (52.9)	8 (47.1)	

The number of women response's evaluated (n = 111) differs from the number receiving chemotherapy (n = 112) because one patient died from neutropenic sepsis after the first cycle of chemotherapy. *The number of women evaluated (n = 109) differs from the total (n = 111), due to an insufficient amount of DNA to perform genotyping by the multiplex polymerase chain reaction (PCR) method; (n): number of patients; *p* values were calculated using the Chi square/Fisher exact test.
